# Structural Diversity of Streptococcal Mutans Synthesized under Different Culture and Environmental Conditions and Its Effect on Mutanase Synthesi

**DOI:** 10.3390/molecules171011800

**Published:** 2012-10-09

**Authors:** Adrian Wiater, Małgorzata Pleszczyńska, Katarzyna Próchniak, Janusz Szczodrak

**Affiliations:** Department of Industrial Microbiology, Maria Curie-Skłodowska University, Akademicka 19, 20-033 Lublin, Poland; Email: mplesz@poczta.onet.pl (M.P.); kasiaproch@poczta.onet.pl (K.P.); szczo@poczta.umcs.lublin.pl (J.S.)

**Keywords:** mutan, glucosyltransferase, cariogenic streptococci, NMR spectroscopy

## Abstract

Streptococcal mutans synthesized under different conditions by growing cultures or by their glucosyltransferases were shown to exhibit a great structural and property diversity. Culturing and environmental factors causing structural differences in mutans were specified. All of the obtained biopolymers (76 samples) were water-insoluble and most of them (72) had a structure with a predominance of α-(1→3)-linked glucose (*i.e.*, the content of α-(1→3)-linkages in the glucan was always higher than 50%, but did not exceed 76%). An exception were four glucans containing more than 50% of α-(1→6)-sequences. In these structurally unique mutans, the ratio of α-(1→3)- to α-(1→6)-bonds ranged from 0.75 to 0.97. Aside from one polymer, all others had a heavily branched structures and differed in the number of α-(1→3), α-(1→6), and α-(1→3,6) linkages and their mutual proportion. The induction of mutanase production in shaken flask cultures of *Trichoderma harzianum* by the structurally diverse mutans resulted in enzyme activities ranging from 0.144 to 1.051 U/mL. No statistical correlation was found between the total percentage content of α-(1→3)-linkages in the α-glucan and mutanase activity. Thus, despite biosynthetic differences causing structural variation in the mutans, it did not matter which mutan structures were used to induce mutanase production.

## 1. Introduction

Mutans, *i.e.*, mixed-linkage [α-(1→3), α-(1→6)] water-insoluble D-glucans, are structural and functional components of cariogenic biofilms and are commonly used as specific and efficient inducers of mutan-degrading enzymes known as mutanases—α-(1→3)-glucan 3-glucanohydrolases [1]. Mutans are synthesized from sucrose by the action of one or more constitutive glucosyltransferases (Gtfs) secreted by mutans streptococci (mainly *Streptococcus mutans* and *S. sobrinus*). Each enzyme has distinctive properties, varying in its requirement for a primer molecule, the proportion of α-(1→3)- and α-(1→6)-linkages, the degree of branching it introduces into the glucan, and the total length of the glucan chain produced. Thus, the overall properties of a mutan depend on the relative activity of different Gtfs and also on their dynamic interactions, since one Gtf may modify the product of another [2,3,4]. Consequently, mutans have a highly branched and diverse structure in which α-(1→3)-glucosidic bonds predominate.

One of the methods of preventing dental caries is to destroy the mutan framework of dental plaque using mutanases synthesized by various bacteria and filamentous fungi [5,6]. However, streptococcal synthesis of mutan for the specific induction and extracellular secretion of mutanase in microbial cultures has only been carried out on a small laboratory scale, and this type of glucan has not yet been made available as a commercial product [7]. Thus, the investigation of the structural diversity of mutans synthesized under changing culture and environmental conditions and the estimation of the effect of these diverse inducers on mutanase activity would facilitate the production of this valuable enzyme. 

Although there are many reports on the linkage analysis of mutans [8,9,10] and their enzymatic degradation [11], most of these studies have been performed on glucans synthesized by isolated and highly purified Gtfs [12,13,14]. Structural information on mutans formed by crude Gtf preparations is sparse [15,16]. On the other hand, data from *in vitro* studies using individual Gtfs indicate that the polymers thus synthesized possess a distinct structure and properties than the products formed by a mixture of Gtfs or a crude enzyme preparation [13,17,18]. In others words, separate enzymes do not mimic the Gtf interactions present in a Gtf mixture, which could modify the final polymer. Therefore, in the present study mutans were produced using non-purified enzymatic preparations containing Gtf complexes which, as showed by Guggenheim [15], Ebisu and Misaki [19], as well as Inoue and co-workers [17], lead to obtaining natural products more resistant to enzymatic digestion, and synthesized in the absence of primer dextran. 

There are many papers on the structure and function of streptococcal mutans [1,3,16], but only a few of them discuss the high degree of structural heterogeneity of these biopolymers synthesized *in vitro* by Gtfs under different environmental conditions [20,21,22]. It should also be noted that the precise causes of the great diversity of water-insoluble α-(1→3)-glucans have not been thoroughly investigated. Moreover, we know of no work on the effect of the diverse structure of the mutans used in microbial cultures as mutanase inducers on the enzymatic activity of this catalyst. Accordingly, the aim of these investigations was to show structural variations in streptococcal mutans caused by their synthesis under different culture and environmental conditions, and to study the influence of structurally diverse biopolymers on the induction of synthesis of *T. harzianum* mutanase.

## 2. Results and Discussion

The production of mutan by cariogenic streptococci involves two separate stages: synthesis and secretion of Gtfs into the culture medium and formation of glucan from sucrose by the same enzymes present in the post-culture supernatant. In a previous study, a detailed chemical structure of a mutan synthesized by cariogenic streptococci was analyzed, and the best operating conditions for efficient production of this polymer were standardized [7,16]. Here, it was assumed that each deviation from optimal conditions estimated for both stages of mutan production can modify the mutual proportions of the different types of links in the polymer leading to a high structural diversity of the glucans obtained in this way. Therefore, the type and the proportions of the main linkages in the mutan macromolecule formed under different culturing and environmental conditions were studied. These conditions were determined using various operating factors with a relatively wide range of action on the individual stages of mutan biosynthesis. 

### 2.1. Structure and Some Properties of Mutans Synthesized by Cariogenic Streptococci Grown under Different Culture Conditions

**Figure 1:**
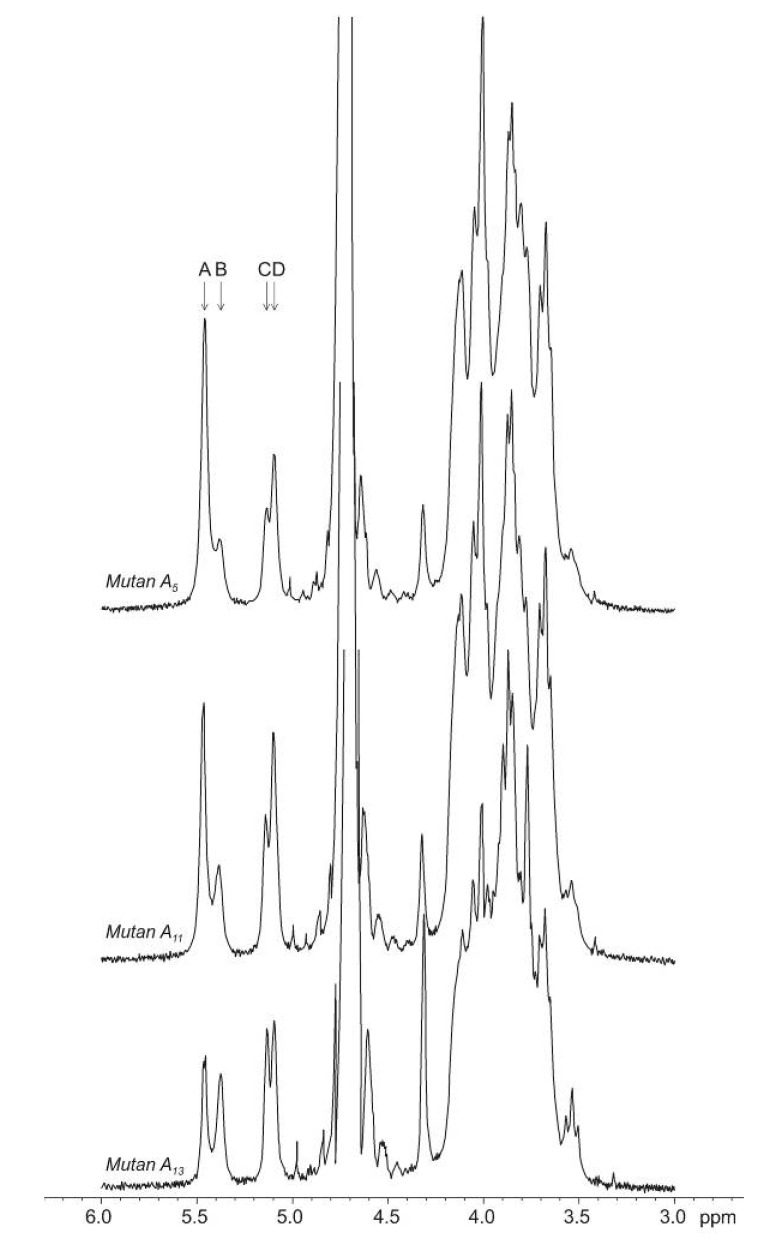
^1^H-NMR spectra of selected streptococcal mutans. Anomeric signals of: (**A**) α-(1→3)-linked glucose; (**B**) α-(1→3)-linked glucose substituted at *O*-6; (**C**) α-(1→6)-linked glucose substituted at *O*-3; (**D**) α-(1→6)-linked glucose. Samples were dissolved in 30% NaOD in D_2_O and spectra were recorded at 300 MHz at 60 °C.

The data summarized in Table 1 clearly indicate that the percentage content of the different types of linkages (calculated from an integration of the anomeric proton signal areas as shown in Figure 1) in the mutan macromolecule and their mutual proportions varied widely and depended on the strain of cariogenic bacteria used to produce Gtfs and the type of medium utilized for the cultivation of mutans streptococci. However, no direct correlations between mutan structure and conditions of mutan production were found. Among the four cariogenic streptococci, the strain *S. sobrinus*/*downei* 21020 and, in most cases, the media I, BHI, TTY, and THB were the best for the production of mutans with the highest content of α-(1→3)-linkages and the highest proportion of (1→3)- to (1→6)-linkages. Experimental results reported earlier by Nisizawa *et al.* [23] also show some strain-dependent differences in the linkage structure of water-insoluble glucans (IG-1) formed by 12 various cultures of *S*. *mutans* cultivated in a sucrose-containing medium. The content of α-(1→3)- and α-(1→6)-linkages in polysaccharide preparations produced by the individual strains of oral streptococci in that study ranged from 49% to 64% and from 31.5% to 49.1%, respectively, so their mutual proportions oscillated between 1.24 and 1.80. The linkage structure and some properties of water-insoluble glucans obtained from 18 oral *S*. *salivarius* isolates were reported by Eifuku and co-workers [24]. Those authors showed that percentage contents of α-(1→3)- and α-(1→6)-linked D-glucosyl residues and their proportions were dependent on the individual strain and differed greatly among the tested cultures.

In the present series of experiments, a total of 20 structurally diverse glucans were obtained. A predominance of (1→3)-linked α-glucans was observed, but no preparations without α-(1→6) glucose-containing chains were detected. All polymers (labeled as group A) were water-insoluble, and most of them (17) had a linkage structure in which the total content of α-(1→3)-glucosidic bonds was higher than 50%. An exception were three glucans (A_13_, A_16_, and A_17_) which contained more than 50% glucose linked by (1→6) glycosidic bonds. In these atypical mutans, the ratio of α-(1→3)- to α-(1→6)-bonds ranged from 0.82 to 0.97, and they were all formed by *S. sobrinus* strain 6070 cultivated in three different media (I, TTY, and THB). Based on the structure of these three unique polymers, it can be speculated that their water insolubility depends not only on the number of α-(1→3)-linkages but also on other factors such as van der Waals or proton interactions leading to the formation of large and stable macromolecular aggregates (supramolecular structures) with a lowered solubility. This supposition finds its confirmation in studies carried out by Yui and co-workers [10]. Those authors found, by checking typical helix models, that formation of hydrogen bonds involving side residues was a major cause of structural stabilization, increasing the insolubility of the highly branching cariogenic α-(1→3)-glucan. Also Nisizawa and co-workers [23] obtained from various strains of oral streptococci a few insoluble glucans in which α-(1→6)-glucosidic bonds predominated. Those authors suggested that the solubility of these biopolymers depended not only upon their content of α-(1→3)-linkages but also upon some additional factors such as molecular weight or other structural features. 

**Table 1:** Structure and some properties of mutans synthesized by cariogenic streptococcal strains cultivated in various media ^a,b^.

Strain	Medium	Mutan ^c^	Content of glucosidic linkages (mol%)				Viscosity (mPa-s)	Optical rotation 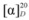 (°)
			α-(1→3) chain		α-(1→6) chain			
			α-(1→3)	α-(1→3,6) ^d^	α-(1→6)	α-(1→3,6) ^e^		
*S. sobrinus*/*downei* 21020	I	A_1_	38.7	19.7	22.1	19.5	8.3	+218
	II	A_2_	44.0	11.9	26.4	17.7	15.8	+208
	BHI	A_3_	44.5	17.8	22.6	15.1	9.7	+214
	TTY	A_4_	48.7	14.0	24.9	12.4	17.8	+216
	THB	A_5_	49.1	14.0	24.5	12.4	6.6	+208
	TSB	A_6_	35.7	19.6	27.7	17.0	6.8	+208
*S. sobrinus *20381	I	A_7_	33.8	23.0	24.0	19.2	8.3	+224
	II	A_8_	34.0	17.2	34.0	14.8	5.3	+206
	BHI	A_9_	35.1	19.1	29.0	16.8	9.3	+144
	TTY	A_10_	36.2	23.7	20.8	19.3	10.2	+210
	THB	A_11_	37.2	16.9	30.5	15.4	7.6	+216
	TSB	A_12_	33.0	22.0	24.7	20.3	8.2	+214
*S. sobrinus *6070	I	A_13_	23.7	25.5	27.8	23.0	4.8	+198
	II	A_14_	28.7	25.7	22.7	22.9	25.4	+224
	BHI	A_15_	38.5	12.7	31.9	16.9	7.1	+110
	TTY	A_16_	32.4	14.5	38.7	14.4	19.9	+112
	THB	A_17_	26.7	18.4	33.9	21.0	6.3	+208
	TSB	- ^f^	-	-	-	-	-	-
*S. mutans *6067	I	A_18_	37.5	14.0	34.2	14.3	13.2	+194
	II	-	-	-	-	-	-	-
	BHI	A_19_	36.9	13.7	32.7	16.7	9.6	+196
	TTY	-	-	-	-	-	-	-
	THB	A_20_	54.1	0.0	45.9	0.0	3.1	+174
	TSB	-	-	-	-	-	-	-

^a^ Media: I, Quivey and Kriger [25]; II, Fuglsang *et al.* [26]; BHI, Brain Heart Infusion; TTY, Hamada and Torii [27]; THB, Todd Hewitt Broth; TSB, Tripticase Soy Broth. ^b^ Culture conditions: medium, 400 mL; temperature, 37 °C; cultivation time, 24 h; aerobic conditions. ^c^ Mutan synthesis conditions: culture supernate, 400 mL, pH-value, not regulated; sucrose, 3%; NaN_3_, 0.05%; temperature, 37 °C; reaction time, 24 h; static conditions. ^d^ Position 6 is a branching point. ^e^ Position 3 is a branching point. ^f^ Not detected. *Note*. The mean of triplicate experiments is shown. Standard deviations (not shown) between the values obtained in each experiment for glucosidic linkages content, viscosity, and optical rotation were less than 3%.

Except for mutan A_20_, all remaining polymers had a highly branched structure. The highest percentage content of α-(1→3)-linkages (about 63%) was found in mutans A_3_, A_4_, and A_5_, which were synthesized by the strain of *S. sobrinus*/*downei* 21020 grown in the BHI, TTY, and THB media. The proportion of (1→3)- to (1→6)- linkages in the structure of these biopolymers was about 1.7. Some strains of mutans streptococci (*S. sobrinus* 6070 and *S. mutans* 6067) grown in TSB, II, and TTY media did not produce any mutan. The great structural diversity of the investigated mutans also had an effect on their physical properties. For example, the values of viscosity and specific optical rotation obtained for those mutans were diversified and oscillated between 3.1 mPa·s and 25.4 mPa·s (glucans A_20_ and A_14_), and between 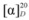 +110° and 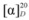 +224° (mutans A_15_ and A_7_).

As it has been shown in our previous report [7], the strain *S. sobrinus*/*downei* 21020 cultivated in medium I produced the largest amount of native mutan, with a yield much higher than any previously reported in the literature. Here, this mutan (A_1_) was characterized by a high content of total α-(1→3)-linkages (58.4%), and a (1.4 times) larger proportion of α-(1→3)- compared to α-(1→6)-linkages. Hence, the strain *S. sobrinus*/*downei* 21020 and medium I were chosen for further studies in which other culturing parameters affecting the structure of mutan were evaluated.

Table 2 shows the structure and some properties of mutans produced by the selected streptococcal strain grown in medium I under different culture conditions. These conditions were established by changing those operating parameters (initial medium pH, temperature, time of cultivation, glucose concentration in the medium, and type of culture) which have a relatively wide range of action on mutan formation. Depending on the culturing factor used, five groups of glucans (designated as B, C, D, E, and F), comprising 23 polymers, were obtained. Those glucans differed widely in their structure and properties. Although all of them were water-insoluble, and had a highly branched structure containing much more than 50% of α-(1→3)-linked glucosyl residues, they showed a moderate structural dissimilarity and differed in the number of the analyzed linkages and their mutual proportion. However, no strict correlations between the culturing factors used and the amount or the proportion of particular linkages in the mutan molecule were found. The highest percentage content of α-(1→3)-bonds (73.4%) and the highest ratio of α-(1→3)- to α-(1→6)-linkages (about 2.8) were found in mutan D_6_ synthesized by streptococci grown in a medium containing 1% of glucose. The lowest values of these structural variables (54.5% and 1.2, respectively) were recorded for mutan E_2_. Walker and co-workers [28,29] studied the effect of a variety of growth conditions on extracellular glucosyltransferase activities of *S*. *mutans* strains in continuous culture. The authors showed that each variation in the relative activities of the particular Gtfs resulted in such a diversity of glucans synthesized from sucrose that structural analysis of those glucans was virtually meaningless without specifying the conditions of growth of the organism by which they were synthesized.

**Table 2:** Structure and some properties of mutans produced under different culture conditions by *S. sobrinus*/*downei* 21020 grown in medium I.

Factor varied ^a,b^	Mutan ^c^	Content of glucosidic linkages (mol%)				Viscosity (mPa-s)	Optical rotation 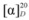 (°)
		α-(1→3) chain		α-(1→6) chain			
		α-(1→3)	α-(1→3,6) ^d^	α-(1→6)	α-(1→3,6) ^e^		
Initial pH of the medium							
6.0	B_1_	56.6	11.7	20.0	11.7	11.7	+184
6.5	B_2_	43.6	15.8	26.0	14.6	18.5	+242
7.0	B_3_	43.8	16.1	26.0	14.1	16.1	+220
7.5	B_4_	38.5	17.8	26.8	16.9	15.5	+222
8.0	B_5_	41.4	17.9	24.0	16.7	13.5	+220
8.5	B_6_	57.9	9.5	23.2	9.4	21.0	+240
Culture temperature (°C)							
30	C_1_	62.9	3.9	27.8	5.4	5.0	+198
37	C_2_	48.0	12.9	27.1	12.0	17.0	+196
40	C_3_	56.0	8.9	24.4	10.7	19.3	+210
Glucose concentration (%)							
0.00	D_1_	43.0	15.8	25.6	15.6	5.4	+148
0.05	D_2_	49.6	17.9	17.1	15.4	8.1	+210
0.10	D_3_	42.7	17.3	23.5	16.5	10.5	+148
0.25	D_4_	48.9	14.8	24.7	11.6	3.8	+152
0.50	D_5_	57.5	14.9	15.6	12.0	5.9	+146
1.00	D_6_	59.8	13.6	14.4	12.4	5.7	+218
Cultivation time (h)							
6	E_1_	57.6	7.2	27.3	7.9	22.9	+260
12	E_2_	41.3	13.2	33.4	12.1	11.2	+230
18	E_3_	33.7	22.2	23.3	20.8	6.9	+218
24	E_4_	34.1	24.4	18.2	23.3	3.9	+206
36	E_5_	43.2	21.1	16.8	18.9	2.4	+208
48	E_6_	42.4	21.5	16.0	20.1	2.3	+202
Kind of culture:							
Anaerobic ^f^	F_1_	43.5	16.1	25.7	14.6	7.7	+222
aerobic	F_2_	34.8	24.3	19.4	21.5	4.5	+210

^a^ Except for the factor that varied as indicated, all other culture and environmental conditions affecting respective stages of efficient mutan production had been standardized earlier [7] and applied here as optimal. ^b^ Optimal culture conditions: medium pH, 7.5; temperature, 37 °C; glucose concentration, 0.1%; cultivation time, 30 h; aerobic conditions. ^c^ Optimal conditions for mutan synthesis: culture supernate pH, 6.0; sucrose, 15%; NaN_3_, 0.05%; temperature, 37 °C; reaction time, 36 h; static conditions. ^d,e^ See Table 1. ^f^ The culture was run in a microbial anaerostat. *Note*. See Table 1.

In the present study, the streptococcal mutans obtained under different culture conditions were also tested for their viscosity and optical rotation. The values of these parameters ranged from 2.3 mPa∙s to 22.9 mPa∙s (glucans E_6_ and E_1_) and from 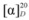 +148° to 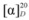 +260° (polymers D_1_ and E_1_), respectively.

### 2.2. Structure and Some Properties of Mutans Synthesized in Post-Culture Supernates Incubated under Different Environmental Conditions

The mixture of glucosyltransferases present in the supernatant fluid obtained after cultivation of the cariogenic strain *S. sobrinus*/*downei* 21020 was subjected to different environmental conditions influencing the formation of streptococcal mutan (Table 3). Depending on basic glucan production variables, *i.e.*, pH, temperature, reaction time, sucrose source, and its concentration, five groups of mutans (designated as G, H, I, J, and K), comprising 33 structurally diversified polymers, were obtained.

**Table 3:** Structure and some properties of mutans formed by streptococcal glucosyltransferases in post-culture supernates incubated under different environmental conditions.

Factor varied ^a^	Mutan ^b^	Content of glucosidic linkages (mol%)				Viscosity (mPa-s)	Optical rotation 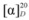 (°)
		α-(1→3) chain		α-(1→6) chain			
		α-(1→3)	α-(1→3,6) ^c^	α-(1→6)	α-(1→3,6) ^d^		
pH of culture supernate							
5.0	G_1_	26.4	24.2	26.4	23.0	4.9	+208
5.5	G_2_	28.2	22.4	26.5	22.9	5.3	+210
6.0	G_3_	31.1	23.9	22.1	22.9	5.6	+210
6.5	G_4_	33.6	23.5	19.2	23.7	6.0	+216
7.0	G_5_	39.2	21.1	18.6	21.1	23.9	+226
7.5	G_6_	45.0	19.5	15.3	20.2	19.6	+220
8.0	G_7_	60.3	15.0	11.8	12.9	30.2	+232
Reaction temperature (°C)							
20	H_1_	17.3	25.7	32.0	25.0	7.1	+184
30	H_2_	27.7	23.9	26.2	22.2	6.6	+206
37	H_3_	32.5	22.7	23.8	21.0	5.7	+218
40	H_4_	35.5	21.9	22.4	20.2	6.0	+214
45	H_5_	52.9	14.8	18.7	13.6	15.2	+234
50	H_6_	51.0	12.9	23.9	12.2	15.5	+240
Sucrose concentration (%) ^e^							
1	I_1_	45.4	20.7	16.3	17.6	4.9	+212
2	I_2_	43.4	21.9	16.6	18.1	4.4	+212
3	I_3_	40.8	22.2	18.0	19.0	4.9	+208
5	I_4_	36.9	22.6	20.4	20.1	5.1	+198
10	I_5_	31.2	23.4	23.6	21.8	4.9	+192
15	I_6_	30.0	23.2	25.3	21.5	5.8	+194
20	I_7_	30.3	23.1	26.1	20.5	5.3	+202
Reaction time (h)							
6	J_1_	31.0	21.6	26.9	20.5	11.8	+216
12	J_2_	33.2	20.7	26.6	19.5	11.1	+220
18	J_3_	34.2	20.8	25.6	19.4	9.4	+218
24	J_4_	33.8	20.5	26.1	19.6	9.1	+218
36	J_5_	37.2	19.7	24.8	18.3	7.5	+218
48	J_6_	38.3	19.4	24.4	17.9	6.8	+214
Sucrose source ^f^							
I	K_1_	30.1	22.9	21.5	25.5	5.7	+210
II	K_2_	30.0	22.9	24.9	22.2	5.1	+200
III	K_3_	30.1	22.7	25.0	22.2	5.3	+212
IV	K_4_	27.4	24.2	25.4	23.0	5.9	+212
V	K_5_	31.7	22.2	23.9	22.2	5.8	+192
VI	K_6_	32.6	21.7	22.2	23.5	5.9	+212
VII	K_7_	30.6	22.2	24.5	22.7	5.7	+210

^a^ Except for the factor that varied as indicated, all other environmental conditions for efficient mutan production in post-culture liquids had been standardized earlier [7] and applied here as optimal. ^b^ Optimal conditions for mutan synthesis: culture supernate pH, 6.0; sucrose, 15%; NaN_3_, 0.05%; temperature, 37 °C; reaction time, 36 h; static conditions. ^c,d^ See Table 1(^d,e^). ^e^ Analytical grade reagent. ^f^ Beet sugar; granulated sugar as a substitute for pure sucrose coming from the following Polish sugar factories: Strzyżów (I), Wróblin (II), Świdnica (III), Glinojeck (IV), Krasnystaw (V), Cerekiew (VI), and Małoszyn (VII). *Note*. See Table 1.

All mutans were water-insoluble and had a highly branched structure. Almost all of them (32) had a linkage structure with a predominance of α-(1→3)-glucosidic bonds (*i.e.*, their content was higher than 50%). An exception was the water-insoluble glucan H_1_, synthesized at 20 °C, which contained more than 50% of α-(1→6)-links. In this structurally unique mutan, the ratio of α-(1→3)- to α-(1→6)-linkages was 0.75.

The examined biopolymers showed a moderate structural dissimilarity and differed in the number of the analyzed linkages and their mutual proportion. Though not statistically significant, some correlations between the changing conditions of mutan synthesis and the amount or the proportion of particular linkages in the polymer molecule were observed. The total percentage content of α-(1→3)-glycosidic bonds and the ratio of the total amount of α-(1→3)- to α-(1→6)-linkages increased gradually along with an increase in supernate pH, reaction time, and temperature. By contrast, an increase in the concentration of sucrose in the reaction mixture lowered the total content and mutual proportion of the two linkages in the mutan molecule. On the other hand, replacement of pure sucrose with the much cheaper granulated sugar beet coming from various sugar factories had no major effect on the linkage structure of the mutan formed. The highest percentage content of α-(1→3)-links (75.3) and the highest ratio of α-(1→3)- to α-(1→6)-linkages (about 3.05) was recorded for mutan G_7_ obtained in a reaction mixture adjusted to pH 8.0. Among the biopolymers obtained in this series of experiments, mutan G_7_ also showed the highest viscosity (30.2 mPa·s) and had a relatively high positive optical rotation (+232°).

The diverse environmental conditions used for enzymatic formation of mutan influenced not only the structure but also the physical properties of the biopolymer. For example, the values of viscosity and specific optical rotation obtained for the analyzed mutans were diversified and ranged from 4.4 mPa·s to 30.2 mPa·s (glucans I_2_ and G_7_) and from 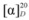 +184° to 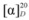 +240° (mutans H_1_ and H_6_), respectively. Data concerning the intrinsic viscosity and the optical rotation of mutans are rarely given in the literature. The values of these parameters provided by some authors [19,30,31] for water-insoluble biopolymers synthesized under optimal conditions by various strains of oral streptococci are relatively high and fluctuate within the limits of 0.46–9.6 [η]_20_ dL/g and from 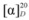 +197° to 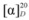 +228°, respectively. As showed by Tsumuraya and Misaki [31], the high positive values of optical rotation obtained for streptococcal mutans are indicative of α-D-glucosidic linkages. Also, Rees and Scott [32] have provided evidence that α-(1→3) linkages in the main chain of glucan confer rigidity and hence high viscosity.

### 2.3. Effect of Structurally Diverse Mutans on Mutanase Synthesis Produced by T. harzianum

Mutan is known as one of the most effective inducers of mutanase synthesis [6,7]. In the chemical structure of this polymeric homoglucan, only α-(1→3)-linkages can be responsible for specific induction of mutanase. Hence, it seems that the polymers with greater numbers of α-(1→3)-links should be much more effective mutanase inducers than the ones with a low content of these bonds. In this context, it is also interesting to know whether there is a close relationship between the content of α-(1→3)-linkages and the activity of fungal mutanase. To answer those questions, 76 structurally diverse mutans, obtained in previous experiments, were used as sole carbon and energy sources for the induction of mutanase production by *T. harzianum* in shaken flask cultures. The content of α-(1→3)-linkages in the particular glucans ranged from 43% (mutan H_1_) to over 75% (mutan G_7_). The inductive effect of mutans on mutanase synthesis was measured as enzymatic activity of supernate obtained after 3 days of fungus cultivation. 

**Figure 2:**
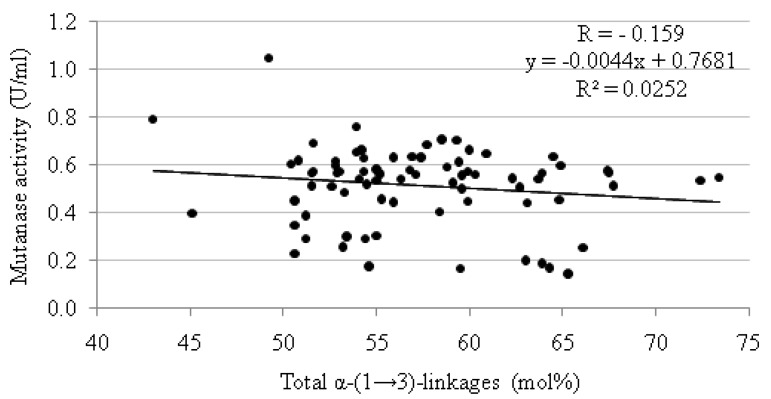
Relationship between mutanase activity obtained on particular mutans as enzyme inducers (76 samples) and the total content of α-(1→3)-linkages in each of these polymers (for details see Table 4). The data obtained from statistical analysis: Pearson correlation (R), determination (R^2^), and linear regression (y), *p < *0.05.

As it can be seen from the data summarized in Table 4, the best inducers of mutanase production were mutans A_13_, H_1_, and K_5_, for which the enzyme reached after 3 days of cultivation the activity of 1.051, 0.792, and 0.761 U/mL, respectively. High mutanolytic activities (0.600–0.707 U/mL) were also at the same time found for 18 other glucans. For comparison, the lowest level of enzyme induction (0.144–0.188 U/mL) was recorded for mutans I_2_, I_4_, I_5_, E_5_, and E_6_. The data obtained from statistical analysis (Pearson correlation coefficient R = −0.159, determination coefficient R^2^ = 0.0252, linear regression y = −0.0044x + 0.7681, *p < 0.05*) indicate a lack of correlation between the total percentage content of α-(1→3)-linkages in the mutan molecule and the mutanase activity induced by it (Figure 2). Thus, despite biosynthetic differences causing structural variation in the mutans, it did not matter which mutan structures were used to induce mutanase production. Based on these results, it can be supposed that the level of mutanase production (expressed as its activity) depends not only on the number of α-(1→3)-linkages in the mutan molecule but also on other factors such as the spatial arrangement of the linkages in the inducer molecule and the accessibility of the enzyme to α-(1→3) sequences. This supposition is confirmed by the fact that in some cases (Table 4) the highest activities of mutanase were achieved for polymers with a low content of α-(1→3)-linkages (e.g., glucans A_13_ and H_1_). In other cases, a relatively high content of α-(1→3)-bonds in a mutan induced a low mutanolytic activity (e.g., polymers I_2_, E_5_, and E_6_).

**Table 4:** Influence of structurally diversified mutans on mutanase production by *T. harzianum* F-340 in shaken flask cultures.

Mutan		Mutanase activity(U/mL)	Mutan		Mutanase activity(U/mL)	Mutan		Mutanase activity(U/mL)
N°	Total content of α-(1→3)-linkages (mol%)		N°	Total content of α-(1→3)-linkages (mol%)		N°	Total content of α-(1→3)-linkages (mol%)	
A_1_	58.4	0.406	C_1_	66.8	0.665	H_3_	55.2	0.564
A_2_	55.9	0.445	C_2_	60.9	0.648	H_4_	57.4	0.632
A_3_	62.3	0.545	C_3_	64.9	0.600	H_5_	67.7	0.514
A_4_	62.7	0.507	D_1_	58.8	0.591	H_6_	63.9	0.566
A_5_	63.1	0.441	D_2_	67.5	0.570	I_1_	66.1	0.253
A_6_	55.3	0.458	D_3_	60.0	0.665	I_2_	65.3	0.144
A_7_	56.8	0.579	D_4_	63.7	0.542	I_3_	63.0	0.201
A_8_	51.2	0.293	D_5_	72.4	0.536	I_4_	59.5	0.168
A_9_	54.2	0.665	D_6_	73.4	0.547	I_5_	54.6	0.175
A_10_	59.9	0.446	E_1_	64.8	0.454	I_6_	53.2	0.256
A_11_	54.1	0.542	E_2_	54.5	0.519	I_7_	53.4	0.300
A_12_	55.0	0.535	E_3_	55.9	0.633	J_1_	52.6	0.509
A_13_	49.2	1.051	E_4_	58.5	0.707	J_2_	53.9	0.656
A_14_	54.4	0.291	E_5_	64.3	0.170	J_3_	55.0	0.582
A_15_	51.2	0.389	E_6_	63.9	0.188	J_4_	54.3	0.572
A_16_	46.9	0.402	F_1_	59.6	0.558	J_5_	56.9	0.635
A_17_	45.1	0.396	F_2_	59.1	0.527	J_6_	57.7	0.686
A_18_	51.5	0.514	G_1_	50.6	0.347	K_1_	53.0	0.573
A_19_	50.6	0.452	G_2_	50.6	0.230	K_2_	52.9	0.566
A_20_	54.1	0.542	G_3_	55.0	0.302	K_3_	52.8	0.600
B_1_	68.3	0.585	G_4_	57.1	0.560	K_4_	51.6	0.691
B_2_	59.4	0.614	G_5_	60.3	0.562	K_5_	53.9	0.761
B_3_	59.9	0.572	G_6_	64.5	0.634	K_6_	54.3	0.631
B_4_	56.3	0.543	G_7_	75.3	0.667	K_7_	52.8	0.618
B_5_	59.3	0.703	H_1_	43.0	0.792			
B_6_	67.4	0.576	H_2_	51.6	0.573			

^a^ Enzyme activity in culture supernatants was measured after 3 days of submerged cultivation of *T. harzianum* on Mandels optimized medium A containing individual mutans as mutanase inducers (0.3%). *Note*. The mean of triplicate experiments is shown. Standard deviations (not shown) between the values obtained in each experiment for mutanase activity were less than 5%.

It should be noted (data not shown) that mutans with a higher content of α-(1→3)-linkages (64–76 mol%) were more susceptible to enzymatic attack of mutanase than the ones containing smaller amounts of these bonds (43–49 mol%). Similar results were reported in a paper published by Eifuku and co-workers [24], where water-insoluble glucans rich in α-(1→3)-linkages showed a higher susceptibility to hydrolysis by mutanase than by dextranase. 

## 3. Experimental

### 3.1. Microorganisms

The cariogenic streptococci used in this study included *Streptococcus mutans* 6067, *S. sobrinus* 6070 (The Collection of Animal Pathogenic Microorganisms, Brno, Czech Republic), *S. sobrinus* 20381 (formerly *S. mutans* 20381) (The German Collection of Microorganisms, Braunschweig, Germany) and *S. sobrinus*/*downei* CCUG 21020 (formerly *S. mutans* OMZ 176) (The Culture Collection, University of Göteborg, Göteborg, Sweden). *Trichoderma harzianum *strain CCM F-340 (Czech Collection of Microorganisms, Brno, Czech Republic) was used as a starting culture for mutanase induction by various preparations of streptococcal mutan.

### 3.2. Bacterial Growth Conditions

Stock cultures of streptococcal strains were stored at −20 °C in 50% glycerol. Unless otherwise stated, the bacteria were grown aerobically in glucose-containing complex media such as Todd-Hewitt broth (THB), tripticase soy broth (TSB), brain-heart infusion (BHI) (Baltimore Biological Laboratory, Cockeysville, MD, USA), and I, II and TTY as given by Quivey and Kriger [25], Fuglsang and co-workers [26], and Hamada and Torii [27], respectively. The media contained in 500-mL flasks, 400 mL each, were autoclaved for 30 min at 121 °C. A precultured broth (24-h-old, 0.5 v/v%) of bacteria grown at 37 °C in the same media was used for flask inoculation. Batch cultures were run at 37 °C for 24 h under stationary conditions. Bacteria were grown under strictly anaerobic conditions in a microbial anaerostat (anaeroJar AG 025A, Oxoid, Basingstoke, UK). Some culturing parameters, listed in Table 2, were evaluated as potential factors modifying the structure of mutans. This Table also gives other experimental details. 

### 3.3. Production of Mutan

Insoluble mutans were synthesized from sucrose by a mixture of extracellular glucosyltransferases present in the post-culture fluids of cariogenic streptococci. The bacterial biomass was separated by centrifugation at 12,000 ×*g* for 20 min. Unless otherwise stated, clear supernatant fluid (without pH adjustment) was allowed to react with sucrose (3%) in the presence of 0.05% sodium azide as a preservative. The water-insoluble glucan formed after incubation at 37 °C for 24 h (henceforth referred to as mutan) was collected by centrifugation at 12,000 ×*g* for 20 min, washed thoroughly with deionized water, and freeze-dried. Some of the important factors influencing the structural diversity of the mutan synthesized in the post-culture supernate as well as other methodological details are specified in Table 3.

### 3.4. Trichoderma harzianum Cultivation

Stock cultures of *T. harzianum*, maintained at 4 °C on potato dextrose agar slants, were used for inoculations. Liquid medium A (pH 5.3), as described by Mandels and co-workers [33], supplemented with 0.3% mutan as a mutanase inducer, 0.05% peptone proteose, and 0.1% Tween 80, was used for mutanase production. Shaken cultures were conducted in 500 mL conical flasks containing 100 mL of sterile medium. The flasks were seeded with conidia to a final concentration of about 2 × 10^7^ conidia/mL and placed on an orbital rotary shaker at 300 rpm and 30 °C for 3 days.

### 3.5. Structural Studies

The structural characteristics of mutans were investigated by ^1^H-NMR. Mutans (20 mg) were dissolved in 0.6 mL of 30% NaOD in D_2_O. The spectra of the alkali-soluble glucans were recorded with an Avance (300 MHz) spectrometer (Bruker BioSpin GmbH, Rheinstetten/Karlsruhe, Germany) at 60 °C. ^1^H chemical shifts were estimated using acetone (*δ*_H_ 2.225 ppm) as the internal standard. The approximate amounts of α-(1→3)- and α-(1→6)-glucosidic linkages and those of branched glucose were calculated from an integration of the anomeric proton signal areas [34]. Specific rotation 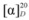 + (*c* 1 M sodium hydroxide) was measured at 589 nm in a Perkin Elmer Automatic Polarimeter (Model 341 LC). The intrinsic viscosity of the polysaccharides (*c* 1 M sodium hydroxide) was measured with a Brookfield (Model DV 3) viscometer at 20 °C. 

### 3.6. Mutanase Assay

The standard mutanase assay mixture contained 0.5 mL of 0.2% (w/v) dextranase-treated mutan (DTM) in 0.2 M sodium acetate buffer (pH 5.5) and 0.5 mL of a suitably diluted enzyme solution. After 1 h incubation at 45 °C, the reducing sugars released were quantified by the Somogyi–Nelson method [35,36]. One unit of mutanase activity (U) was defined as the amount of the enzyme hydrolyzing mutan to yield reducing sugars equivalent to 1 µmol of glucose/min, and expressed as units per mL of culture (U/mL). 1 U corresponds to 16.67 nkat.

### 3.7. Preparation of Dextranase-Treated Mutan (DTM) for Mutanase Activity Determination

DTM was prepared (50 dextranase U/per g of native mutan, pH 6.0, 37 *°*C, 3 × 24 h) and used as a substrate for mutanase activity determination. Native mutan was synthesized from sucrose as described previously [7]. An analysis of the linkage structure of the native and the dextranase-treated mutans, as determined by ^1^H-NMR, showed that they were mixed-linkage α-(1→3) and α-(1→6) biopolymers with a higher proportion of α-(1→3)- than α-(1→6)-linkages, namely, 59.1 and 40.9 mol% for the native mutan and 79.8 and 20.2 mol% for DTM, respectively. 

### 3.8. Statistical Analysis

Statistical analysis of data was performed on three replicates from each treatment. Standard deviations between the values obtained in each experiment for glucosidic linkage content and viscosity and optical rotation were less than 3%, and those for mutanase activity were less than 5%. Standard deviations were determined using Microsoft® Excel 2000 (Microsoft Corp., Redmond, WA, USA). The Pearson correlation coefficient (R), the determination coefficient (R^2^), and the linear regression (y) were determined (using Microsoft® Excel 2000) to show the direction and strength of the relationship between the total percentage content of α-(1→3)-linkages in mutan chains (obtained under different conditions) and mutanase activities in the culture fluid of *T. harzianum* after induction by a particular inductor. Other methodological details are given in legends to Tables. 

## 4. Conclusions

In conclusion, the present study clearly reveals the great structural diversity of streptococcal mutans formed under different conditions by growing cultures and by their glucosyltransferases. Bacterial strain, kind of medium, and differential culture and environmental conditions affect the number of α-(1→3)- and α-(1→6)-linkages in the mutan molecule and their mutual proportion, which results in smaller or larger dissimilarities in cariogenic mutans. However, despite biosynthetic differences causing structural variation in the mutans, it did not matter which mutan structures were used to induce mutanase production. No correlation was found between the total percentage content of α-(1→3)-bonds in a glucan polymer and mutanase activity, but mutans with higher contents of (1→3)-linked glucose had enhanced susceptibility to mutanolysis. It seems beneficial for prospective production of this valuable enzyme since it relieves the producer of the necessity of applying an inducer with a closely defined linkage structure.
